# 3D Printable Biophotopolymers for *in Vivo* Bone Regeneration

**DOI:** 10.3390/ma8063685

**Published:** 2015-06-19

**Authors:** Guenter Russmueller, Robert Liska, Juergen Stampfl, Christian Heller, Andreas Mautner, Karin Macfelda, Barbara Kapeller, Roman Lieber, Agnes Haider, Kathrin Mika, Christian Schopper, Christos Perisanidis, Rudolf Seemann, Doris Moser

**Affiliations:** 1 Department of Cranio-, Maxillofacial and Oral Surgery, Medical University of Vienna, Waehringer Guertel 18-20, 1090 Vienna, Austria; E-Mails: n0742030@students.meduniwien.ac.at (A.H.); n0003271@students.meduniwien.ac.at (K.M.); christian.schopper@meduniwien.ac.at (C.S.); christos.perisanidis@meduniwien.ac.at (C.P.); rudolf.seemann@meduniwien.ac.at (R.S.); doris.moser@meduniwien.ac.at (D.M.); 2 Division of Macromolecular Chemistry, Institute of Applied Synthetic Chemistry, Vienna University of Technology, Getreidemarkt 9, 1060 Vienna, Austria; E-Mails: robert.liska@tuwien.ac.at (R.L.); andreas.mautner@tuwien.ac.at (A.M.); 3 Institute of Materials Science and Technology, Vienna University of Technology, Getreidemarkt 9, 1060 Vienna, Austria; E-Mails: juergen.stampfl@tuwien.ac.at (J.S.); christian.heller@tuwien.ac.at (C.H.); 4 Department of Biomedical Research, Medical University of Vienna, Waehringer Guertel 18-20, 1090 Vienna; E-Mails: karin.macfelda@meduniwien.ac.at (K.M.); barbara.kapeller@meduniwien.ac.at (B.K.); roman.lieber@meduniwien.ac.at (R.L.)

**Keywords:** photopolymers, additive manufacturing technology, bone regeneration, *in vivo*

## Abstract

The present study investigated two novel biophotopolymer classes that are chemically based on non-toxic poly (vinyl alcohol). These vinylesters and vinylcarbonates were compared to standard acrylates *in vitro* on MC3T3-E1 cells and *in vivo* in a small animal model. *In vitro*, both vinylester and vinylcarbonate monomers showed about tenfold less cytotoxicity when compared to acrylates (IC_50_: 2.922 mM and 2.392 mM *vs.* 0.201 mM) and at least threefold higher alkaline phosphatase activity (17.038 and 18.836 *vs.* 5.795, measured at [10 mM]). *In vivo*, polymerized 3D cellular structures were implanted into the distal femoral condyle of 16 New Zealand White Rabbits and were observed for periods from 4 to 12 weeks. New bone formation and bone to implant contact was evaluated by histomorphometry at end of observation. Vinylesters showed similar rates of new bone formation but significantly less (*p* = 0.002) bone to implant contact, when compared to acrylates. In contrast, the implantation of vinylcarbonate based biophotopolymers led to significantly higher rates of newly formed bone (*p* < 0.001) and bone to implant contact (*p* < 0.001). Additionally, distinct signs of polymer degradation could be observed in vinylesters and vinylcarbonates by histology. We conclude, that vinylesters and vinylcarbonates are promising new biophotopolymers, that outmatch available poly(lactic acid) and (meth)acrylate based materials.

## 1. Introduction

The treatment of bone defects remains a challenging problem. In a high number of orthopedic surgical procedures a bone substitute is necessary. Autologous bone grafting is currently the most frequently used method for bone replacement, although key disadvantages as donor site morbidity with prolonged hospitalization, graft resorption or limited shaping of these grafts have not been solved [1,2].

Autologous free bone grafting serves as gold standard and shows good osteoinduction and osteoconduction in the management of smaller bone defects. In larger reconstructions however, it results in poor osseointegration and graft resorption caused by deficient blood supply. Other techniques such as microvascular grafts or distraction osteogenesis appear better suited, but are technically more difficult and sometimes are associated with even more complications [3,4].

In recent years, alternative therapeutic approaches, such as alloplastic bone replacement materials or growth factors have been developed. Among biomaterials currently under investigation one can find ceramics as well as polymers. Available alloplastic bone replacement materials feature unsatisfying biological and mechanical properties. They are inferior to autologous bone and fail to prove in clinical routine [5,6].

Poly(lactic acid) (PLA), show hydrolytic degradation, under which PLA forms acidic groups that induce autocatalytic bulk erosion, leading to uncontrolled loss of mechanical properties. Local decrease of pH value and the release of lactic acid can cause inflammation reactions or even tissue necrosis [7].

(Meth)acrylate-based polymers and their (meth)acrylic groups always show some irritancy and sometimes cytotoxicity. During degradation these residual groups form harmful (meth)acrylic acid. Degradation leads to high molecular (70 kD) poly(meth)acrylic acid that cannot be excreted from the human body and therefore might cause inflammation reactions [8].

In the group of biodegradable polymers, polyesters such as poly(lactic acid) (PLA), poly(glycolic acid) (PGA) or poly(e-caprolactone) (PCL) comprise the earliest and most extensively investigated class of materials and are used in several clinical applications.

Classical polymer processing methods such as extrusion or injection molding are frequently used for processing of these materials to produce sutures, bone fixation materials and other medical devices. Unfortunately, these techniques have very restricted capability for the manufacturing of 3D cellular structures [7,9].

One of the milestones in regenerative medicine has been the development of 3D scaffolds that guide cells to form functional tissue. Recently, layered manufacturing techniques, known as Additive Manufacturing Technology (AMT) or 3D Printing, have been successfully used to fabricate complex scaffolds. Besides Selective Laser Sintering, Stereolithography (SL), inkjet-based techniques that use photopolymerizable formulations are of significant importance [10,11].

Photopolymerization is a widely explored technology that has recently been recognized to also have great potentialities in the biomedical field. Well-established applications are contact lenses or dental filling materials [12,13,14].

The two main advantages of using photopolymer-based AMT, compared to other methods, are the excellent achievable feature resolution and the possibility to tune the mechanical and functional properties over a wide range by modifying the resin. Reactivity, processing viscosity, biocompatibility and mechanical properties and degradation behavior can be set according to the specific requirements [15].

To compensate the given disadvantages of available alloplastic materials, we aimed to develop and test vinyl ester and vinyl carbonate based monomers that are polymerizable by AMT [16,17].

The polymer backbone and final degradation product of these poly(vinyl esters) and poly(vinyl carbonates) upon hydrolysis is poly(vinyl alcohol). This is nontoxic, FDA-approved and well known as a pharmaceutical additive and its use in medical implants and in food industry.

Based on own previous studies investigating poly(vinyl esters) and poly(vinyl carbonates), we selected the most promising monomers according to polymerization and mechanical properties for comprehensive *in vitro* and *in vivo* testing. Standard acrylate-based mono- and polymers served as controls, both *in vitro* and *in vivo* [18,19].

Osteoblast-like murine MC3T3-E1 cells were incubated *in vitro* with selected monomers in different concentrations and cell growth was determined by the Alamar-Blue assay. Additionally, alkaline phosphatase activity was tested *in vitro* as a surrogate for the synthetic activity of the MC3T3-E1 cells.

For *in vivo* studies 3D cellular structures of 5 mm × 5 mm × 8 mm were built by AMT and implanted into surgically created bone defects of 16 New Zealand White Rabbits and observed for 4 to 12 weeks. At end of observation, the animals were sacrificed and the specimens were retrieved for histological and histomorphometric evaluation of the biological behavior of the cellular structures.

## 2. Experimental Section

### 2.1. Monomer Synthesis

#### 2.1.1. Acrylates

For this experimental setting, the acrylate monomers were not synthesized, as they are commercially available agents.

**Figure 1:**
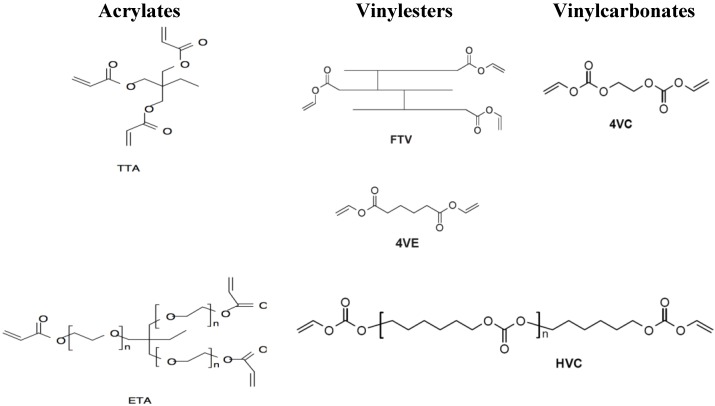
Investigated vinylester and vinylcarbonate monomers and acrylate references. Acrylates: Ethoxylated trimethylolpropane triacrylate (**ETA**) and trimethylolpropane triacrylate (**TTA**); Vinylesters: Adipic acid divinyl ester (**4VE**) and trimer fatty acid vinyl ester (**FTV**); Vinylcarbonates: Ethylene glycol divinyl carbonate (**4VC**) and poly(hexamethylene carbonate) divinyl carbonate (**HVC**).

Ethoxylated trimethylolpropane triacrylate (**ETA**) and trimethylolpropane triacrylate (**TTA**) (both Sigma Life Science, St. Louis, MO, USA) were selected to serve as controls within *in vitro* and *in vivo* experiments (see Figure 1).

#### 2.1.2. Vinylesters

Mercury(II) and hydroquinone as inhibitor were added to a suspension of the appropriate acid in a large excess of vinyl acetate. After stirring under argon atmosphere, p-toluene sulfonic acid was added and the reaction mixture was refluxed for 4–24 h. After cooling the resulting solution was diluted with ethyl acetate and extracted with NaOH.

The organic layer was dried over sodium sulfate and concentrated. The crude product was purified by flash chromatography on silica gel petroleum ether/ethyl acetate.

Based on previous experiments, the monomers adipic acid divinyl ester (**4VE**) and trimer fatty acid vinyl ester (**FTV**) were selected for the presented trial (see Figure 1) for the “vinylester” series [18].

#### 2.1.3. Vinylcarbonates

The most frequently used method for preparation of vinyl carbonates is the conversion of alcohols with vinyl chloroformate. As vinyl chloroformate is commercially available, we chose this route that can convert a large variety of alcohols and primary and secondary amines, to vinyl carbonates and vinyl carbamates in the presence of pyridine or sodium carbonate as acid scavenger.

As representative monomers for vinylcarbonates, ethylene glycol divinyl carbonate (**4VC**) and poly (hexamethylene carbonate) divinyl carbonate (**HVC**) were used for *in vitro* and *in vivo* experiments (see Figure 1) [19].

### 2.2. In Vitro Experiments

The highly differentiated pre-osteoblastic cell line MC3T3-E1 (Subclone 4, ATCC^®^, Catalog No. CRL-2593™) was cultured in DMEM (Sigma Life Science, high glucose, St. Louis, MO, USA) supplemented with 10% FBS (Gibco^®^ by Life Technologies, Carlsbad, NM, USA), 100 IU/mL Penicillin and 100 µg/mL Streptomycin (Gibco^®^ by Life Technologies) at 37 °C with 95% humidity and 5% CO_2_.

Cells were passaged using 0.05% Trypsin-EDTA (Gibco^®^ by Life Technologies) and cultured on 25 cm^2^ Flasks (Corning^®^ Costar^®^).

To determine the effect of monomers on cell viability and alkaline phosphatase activity, cells were seeded onto a 96-well plate (Corning^®^ Costar^®^) with a density of 6400 cells per well and allowed to attach overnight. The next day supernatants were discarded and cells were treated with different concentrations of monomers (10, 5, 2.5, 1.25, 0.63, 0.31 and 0.16 mM).

For better solubility, monomers were diluted in the previously described medium supplemented with 1% (v/v) Dimethylsulphoxide (DMSO Hybri-Max^®^, Sigma Life Science). An untreated control with culture medium and a control with culture medium containing 1% (v/v) DMSO were kept.

After 5 days of cultivation, supernatants were collected and cell viability was determined using the viability reagent AlamarBlue^®^ (Invitrogen^®^ by Life Technologies) following the manufacturer’s instructions. The assay measures the irreversible reaction of resazurin to resorufin, which is proportional to aerobic respiration.

The alkaline phosphatase (ALP) activity was determined from supernatants by using a p-Nitrophenylphosphate assay (NPP, Sigma Life Science) according to the technical bulletin (Procedure No.104, Sigma Life Science).

All *in vitro* experiments were performed in triplicates.

### 2.3. Fabrication of 3D Cellular Structures by AMT

Based on previous results of reactivity measurements and mechanical testing, we selected the following monomer formulations and ratios for production of the 3D cellular structures [18,19]:

- Acrylates: ETA:TTA 1:1
- Vinylesters: 4VE:FTV 3:1
- Vinylcarbonates: 4VC:HVC 4:1

Light penetration tests were performed to determine the amount of initiator and absorber required completely to cure one layer of 50–100 μm thickness. Formulations with 3 wt % of IrgacureVR 819 (BASF Schweiz AG, Basel, Switzerland) as photoinitiator and 0.15 wt % of CGL 097 (BASF Schweiz AG) as UV-absorber showed best results [18].

For the fabrication of 3D cellular structures a lithography-based AMT system was used, which uses dynamic masks based on DLP-technology (digital light processing) for exposing individual layers of a photopolymer [20].

The layers were structured with a light intensity of 800 mW/dm^2^, a layer thickness of 50 μm (acrylates and vinylesters) or 100 μm (vinylcarbonates), respectively, and an exposure time of 2 min per layer (3 min for the first three layers).

After completion of the structuring process the part was rinsed with ethanol and the final parts were obtained after post-curing under a UV lamp to lower the amount of residual monomers.

**Figure 2:**
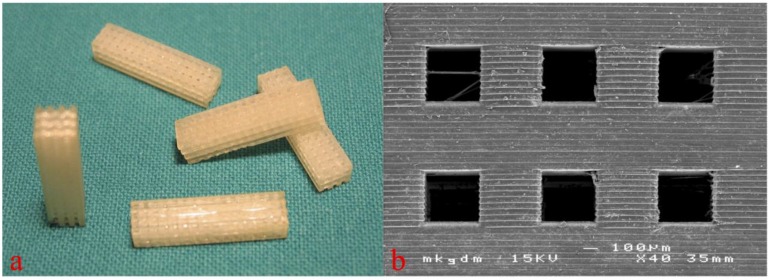
Macroscopic (**a**, figure from [18]) and scanning electron microscopic view (**b**) of the 3D cellular structures for *in vivo* testing. Copyright John Wiley and Sons 2015.

3D cellular structures of 5 mm × 5 mm × 8 mm dimension with pore sizes of 300–500 μm were built by AMT (see Figure 2). Before implantation, the 3D cellular structures built by AMT were gamma sterilized.

### 2.4. In Vivo Experiments and Histologic Sample Preparation

All surgical procedures were performed after approval by the local ethics committee at the animal research facilities of the Department of Biomedical Research, Medical University of Vienna.

Representing a standard animal model for biomedical research, adult female New Zealand White Rabbits (Charles River, Germany), weighing approximately 3 kg were used for *in vivo* testing. The animals were premedicated with an intramuscular injection of 25 mg/kg ketamine and 2 mg/kg xylazine. For anesthetic induction, additional ketamine and xylazine were administered.

After the onset of muscle relaxation, the respective animal underwent orotracheal intubation followed by the insertion of an orotracheal tube. The anesthesia was maintained with volume-controlled ventilation with an oxygen-air mixture plus 1% to 2% isoflurane and the administration of a fentanyl bolus of 0.025 mg. Postoperative analgesia was managed by subcutaneous administration of 0.05 mg/kg buprenorphine every 8 h for 2 days.

**Figure 3:**
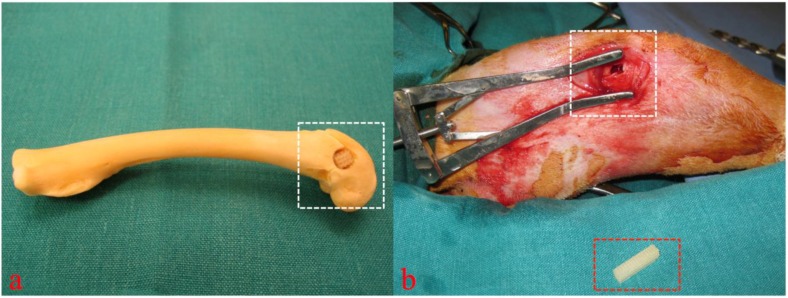
Cadaver simulation (**a**) and intra operative view (**b**, figure from [19]) of implantation site (white rectangular) and 3D cellular structure (red rectangular) before insertion into the drilling defect. Copyright John Wiley and Sons 2015.

After anesthetization of the animals the rear right leg was washed and shaved. Then the layerwise preparation to the lateral side of the distal femoral condyle was carried out followed by splitting the periosteum above the bone. A drilling hole of 5 mm diameter and 10 mm depth was then placed with carbide drills of increasing size until reaching the final diameter avoiding any perforation into the nearby joint (Figure 3) [18,19].

To reach high primary stability of the 3D cellular structures, they were placed into the drilling holes using press-fit. The wound closure was performed again layerwise using absorbable polyglactin and silk sutures. For the time of observation the animals were kept in single stables at the local animal testing facility.

At the end of the particular observation period the animals were euthanized by injection of 25 mg/kg ketamine and 2 mg/kg xylazine, followed by an overdose of phenobarbital (120 mg/kg).

After exitus of the respective animals, a total number of 16 bone samples containing the whole distal femoral bone were explanted and immediately placed in 4.5% buffered formaldehyde solution (pH 7.4) and initially fixed. The specimens were processed undecalcified with a modification of the Donath technique [21].

After fixation, samples were dehydrated in a graded ethanol series (70%–100% ethanol). To ensure good fixation and dewatering of the specimen, all the work steps were carried out with agitating equipment and regular changing of the media. Afterwards samples were transferred to synthetic resin monomer (MMA, methylmetacrylate, Sigma Life Science) for 1 day and then placed into plastic embedding mixture of MMA, nonylphenol polyglycol ether (Sigma Life Science) and benzoyl peroxide (Merck, Darmstadt, Germany) as initiator. Controlled constant polymerization then occurred at room temperature over a period of 7 days followed by a curing phase of 8 days at 37 °C in the incubator.

After hardening of the resin, specimens were trimmed to the appropriate size on a manual grinder and prepared according to the grinding technique for undecalcified hard tissue.

The surfaces to be examined were glued to a plastic microscope slide with a fixation adhesive, divided with a precision disk saw and then ground to the desired thickness of 20 μm using a micro-grinding system, with changing abrasive paper grades (1200, 2400, 4000). To remove any remaining grinding marks the ground surfaces were subsequently polished with velvet disks. The thin sections were then subjected to standardized surface staining for ground specimens (1% thionine stain).

### 2.5. Histologic and Histomorphometric Evaluation

All specimens underwent histomorphometric and histomorphological analysis. After blinding, two cross-sectional slides of each surgical defect, containing the implanted 3D cellular structures, were examined by transmission light microscopy (Eclipse 800, Nikon Corp., Tokyo, Japan) connected to a digital camera (Sony 950 Power HAD, Sony Corp., Tokyo, Japan).

The following were examined: Newly formed bone, sprouting of blood vessels, the turnover of newly formed bone tissue, the processes occurring on the surface of the 3D cellular structures, e.g., bone deposition and potential degradation or surface erosion of the polymer structures.

**Figure 4:**
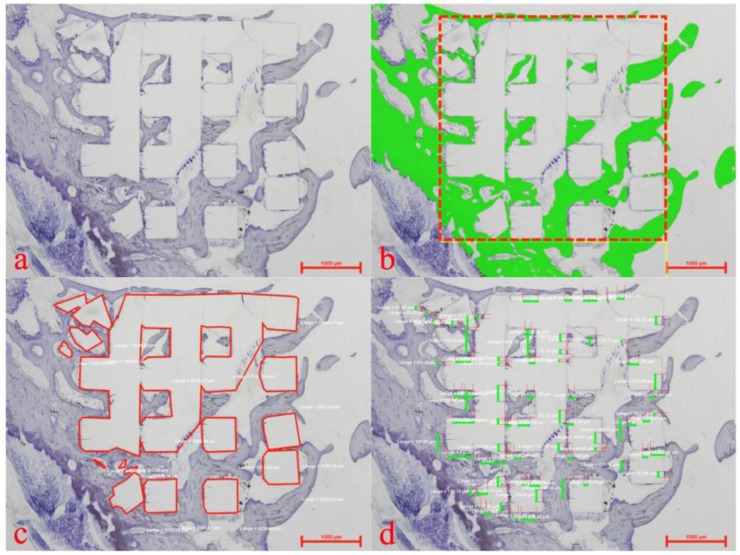
Histomorphometric evaluation showing a plain thionine stain (**a**), the region of interest (**b**) (red rectangular) for assessment of newly formed bone (**b**) (green), the marked extent of the implanted 3D cellular structure (**c**) (red lines) and allocation of bone to implant contact (**d**) (green bars).

Within the whole 3D cellular structure, percentages of newly formed bone and bone to implant contact (BIC) were assessed by histomorphometry (see Figure 4) with a semi-automated image analysis system (NIS- Elements AR 2.3.0, Nikon Corp.).

### 2.6. Statistical Methods

As the Poisson distribution is considered to be best for modeling areas or volumes, Poisson regression models were used to model the new bone formation and the bone to implant contact (BIC) within the scaffold area of the implantation site.

The BIC and the newly formed bone were estimated by the resin as dummy factors (baseline was ETA/TTA) and by 1/time, since the regressands were assumed to asymptotically reach a maximum value over time. Each effect was tested using a Wald’s test.

A *p*-value of <0.05 was defined as significant. All calculations were performed using the statistical programming environment “R” (version 2.15.1, Vienna, Austria).

## 3. Results and Discussion

### 3.1. In Vitro Results

Monomer toxicity reflects one of the central problems of biomedical use of (photo)polymers. To address this issue, the representative monomers of all three groups were tested on MC3T3-E1 cells before *in vivo* application. To determine the effect on cell viability, the cells were incubated with descending monomer concentrations from 10 mM to 0.16 mM.

After five days of cultivation, monomer toxicity was assessed by the colorimetric AlamarBlue^®^ assay by determination of the monomer concentration (IC_50_), at which the activity of resorufin was reduced to 50% of the control. The IC_50_ was then calculated by a dose-response-curve (GrapPad PRISM, GraphPad Software Inc., La Jolla, CA, USA)

By comparing the acrylates to our biophotopolymers vinylesters and vinylcarbonates, the results clearly show the significantly lower monomer toxicity of these new monomers by at least a factor of 10 (see Table 1). These results absolutely concur with our previous data and available literature and underline the postulated superior biological properties of vinylesters and vinylcarbonates [16,17].

**Table 1:** IC_50_ values and alkaline phosphatase (ALP) activity of acrylates, vinylesters, and vinylcarbonates measured *in vitro*.

Monomer	Cell Viability (IC_50_)	95% Confidence Intervals (IC_50_)	ALP-Activity
Acrylates (ETA)	0.201 mM	0.175–0.232	5.795 [10 mM]
Vinylesters (4VE)	2.922 mM	2.533–3.370	17.038 [10 mM]
Vinylcarbonates (4VC)	2.392 mM	1.860–3.075	18.836 [10 mM]

The results retrieved from the AlamarBlue^®^ assay could be proved by the measurement of the alkaline phosphatase (ALP) activity of the MC3T3-E1 cells. ALP catalyzes the hydrolysis of phosphate esters in alkaline buffer and produces an organic radical and inorganic phosphate. ALP activity represents a surrogate for metabolic cell function.

As expected, measured levels of ALP activity were higher than the acrylates for both our new monomers. At the highest monomer concentration of 10 mM, vinylesters and vinylcarbonates showed an approximately three fold higher activity than acrylates (see Table 1). Compared to the controls (cells without monomers), the new monomers showed no difference in ALP activity, even at the highest monomer concentration of 10 mM (data not shown).

Altogether, this lower cytotoxicity of vinylester and vinylcarbonate monomers can be linked to their lack of acrylic double bonds that are very reactive and can easily interfere with cellular proteins or DNA [8].

Vinylesters and vinylcarbonates clearly proved *in vitro* their postulated suitability for biomedical use, as low toxicity of residual monomers is a prerequisite for successful application of these materials *in vivo*.

### 3.2. In Vivo Results

#### 3.2.1. Histology

For assessment of biocompatibility and potential *in vivo* degradation of the biophotopolymers, 3D cellular structures of 5 mm × 5 mm× 8 mm in size were implanted into the distal femoral condyle of New Zealand White Rabbits [18,19].

Stabilization of the 3D cellular structures within the bone defect was reached by press fit placement of these structures. Similar to *in vitro* experiments, acrylates, vinylesters, and vinylcarbonates were evaluated by *in vivo* testing and histology.

**Figure 5:**
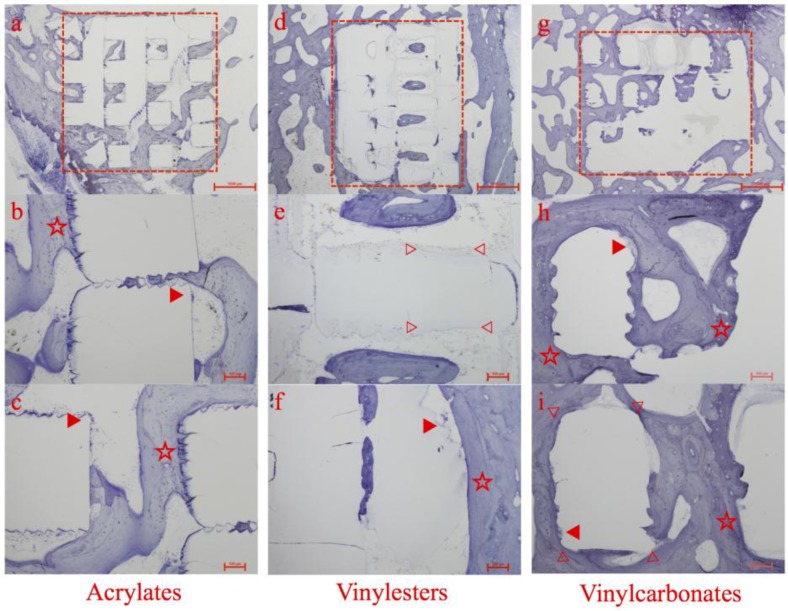
Histologic overview and details of specimens containing acrylates (**a**–**c**), vinylesters (**d**–**f**), and vinyl carbonates (**g**–**i**) as 3D cellular structures (marked as red rectangular in a, d and g) retrieved after 12 weeks of observation. Marked details represent foreign body giant cells (▶), areas of high bone to implant contact (☆) and areas of obvious surface erosion/resorption (▷ ◁).

Histology demonstrated that all three tested materials possess osteoconductive properties, to a greater or lesser extent. In all three groups the 3D cellular structures integrated well into newly formed bone. The polymer material itself could be observed, appearing as residual white matter, not absorbing any thionine stain (see Figure 5a,d,g).

All of the 3D cellular structures maintained their shape and structure; nevertheless signs of surface erosion or disputable resorption were detectable. Additionally, they were shown to be biocompatible and non-toxic. No adverse biological responses occurred. Foreign body giant cells (FBGC) were observed on the surface of the 3D cellular structures in all three experimental groups (see Figure 5, ▶).

The observation of such FBGCs following implantation of a medical device or biomaterial represents the end-stage response of the inflammatory and wound healing process. They release mediators of degradation such as reactive oxygen intermediates, degradative enzymes, and acid into the zone between cell frontier and biomaterial surface, which mediates biomaterial resorption, on condition that the biomaterial shows any capability of resorption.

##### Acrylates

This group, which served as control to vinylesters and vinylcarbonates, showed satisfying histological results in all specimens. Abundant new bone formation, whether directly contacted to the inserted 3D cellular structures or inside the pores, could be regularly observed.

Especially selected regions of the 3D cellular structures exhibiting a microscopic rough and serrated surface featured extraordinary high levels of adherent newly formed bone without any sign of gap between bone and scaffold. This microscopic structure was caused by AMT and reflects bone’s preference for rough surfaces (see Figure 5b,c, ☆).

Numerous foreign body giant cells containing large numbers of nuclei were observed on segments of the surface of the inserted 3D cellular structures (see Figure 5b,c, ▶), but no signs of polymer degradation or resorption could be found. This lack of any decay is in absolute accordance with the chemical nature of the acrylates.

##### Vinylesters

Compared to the control group, the vinylester containing specimens showed a comparable amount and pattern of newly formed bone around the 3D cellular structures. The majority of newly formed trabeculae were composed of lamellar bone with numerous osteoblasts in close apposition to each other localized along the polymer surface (see Figure 5f, ☆). Again, multinucleated foreign body giant cells were present (see Figure 5f, ▶).

New bone formation and consequently bone to implant contact were less distinct inside the 3D cellular structures. Islands of lamellar bone located centrally inside the pore system of the scaffolds without direct bone to implant contact were observed in several specimens (see Figure 5d).

Within this pore system, clear signs of material degradation could have been noticed by the loss of the ruffled polymer surface, that was caused by the layerwise AMT building (see Figure 5e, ▷ ◁). 

Different to the vinylcarbonates, this material degradation was predominately found in surface areas with no bone to implant contact. During this degradation process, whether by enzymes or hydrolysis, no polymer particles were detached from the 3D cellular structures. This could have triggered a severe inflammatory response.

##### Vinylcarbonates

Here, the 3D cellular structures showed the best tissue integrity with direct or close contact between polymer and newly formed bone. The matrix architecture was filled with new bone in combination with islets of fibrous tissue. Again, multinucleated foreign body giant cells were present on the polymer surface (see Figure 5h,i, ▶).

The vinylcarbonates showed the highest amount of newly formed bone in histology. In most specimens, the 3D cellular structure appears to be totally incorporated into the host bone. The surfaces were continuously lined by newly formed bone or fibrous tissue.

The absence of inflammatory cells in the regions between the polymer surface and the bone suggest that the polymer was tolerated well by the surrounding tissue components (see Figure 5h,i, ☆).

Regarding material degradation, the broad loss of the ruffled surface structure, accompanied with high bone to implant contact and thick layer of newly formed bone surrounding the 3D cellular structures, the vinylcarbonates showed the most promising results (see Figure 5i, ▷ ◁).

#### 3.2.2. Histomorphometric Evaluation

After histological assessment, all specimens were processed by histomorphometry. Within the defined region of interest (see Figure 4) area fractions of newly formed bone and the bone to implant contact were determined.

**Figure 6:**
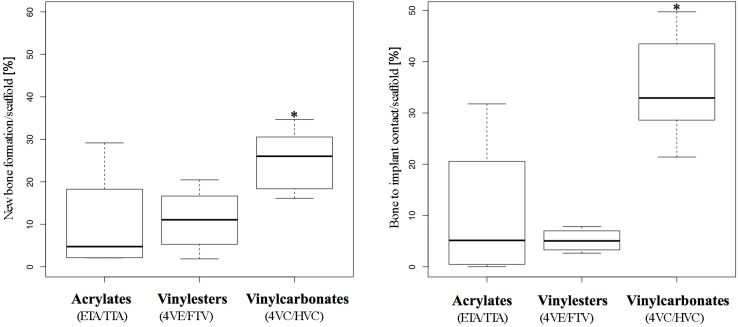
Histomorphometry. Box plot analysis showing 25th, 50th, and 75th percentiles (horizontal bars), and 1.5 interquartile ranges (error bars) of *new bone formation* (left) and *bone to implant contact* (right). Significantly higher values from Poisson regression are *-marked.

The observations and characteristics of the respective photopolymers made in histology could be proved by histomorphometric evaluation, where the vinylcarbonates showed the highest measured values for new bone formation and bone to implant contact (see Figure 6).

##### New Bone Formation

In the Poisson regression model applied, the rates of new bone formation significantly increased over time and asymptotically converged to a statistical peak value in all three groups (see Table 2, “Time model”). This finding matches absolutely with the observations in histology, showing higher amounts of new bone formation in specimens retrieved after the longest observation period.

Compared to the acrylates, which served as control, the vinylesters did not lead to a significantly higher new bone formation. As monomer toxicity can be ruled out as a reason for this finding, the high amount of hydrophobic 4VE within the vinylester resin could be related, as the histological pattern (see Figure 5d) shows a tendency to quite broad gaps between newly formed bone and polymer scaffold.

In contrast, vinylcarbonates showed significantly higher new bone formation in both univariate and multivariate analysis (see Table 2, “Photopolymer model”). This statistical finding is in absolute accordance with histology and reflects the superior biocompatibility of the vinylcarbonates due to monomer toxicity and osteoconductive properties.

**Table 2:** Poisson regression models of new bone formation within the scaffold estimated by photopolymer, time, and both. The first column shows the regressors, the second the estimated coefficients of the univariate models, the third the *p*-values of the univariate Wald’s tests, the fourth the estimates of the multivariate model (BIC = e^intercept + Vinylesters + Vinylcarbonates + 1/time^), and the last column the *p*-values of the Wald’s test of the multivariate model.

Factor	Univariate Estimate [95% CI]	*p*-value of Univariate Model	Multivariate Estimate [95% CI]	*p*-value of Multivariate Model
*“Photopolymer model” of new bone formation*				
Intercept (control) Acrylates	2.32 [2.00; 2.61]	<0.001	2.75 [2.37; 3.10]	<0.001
Vinylesters	0.08 [−0.30; 0.48]	0.68	−0.11 [−0.49; 0.29]	0.60
Vinylcarbonates	0.91 [0.57; 1.27]	<0.001	0.68 [0.34; 1.05]	<0.001
*“Time model” of new bone formation*				
Intercept	3.11 [2.92; 3.32]	<0.001	–	
1/time	−15.46 [−25.77; −7.98]	<0.001	−12.28 [−22.16; −4.99]	0.004

##### Bone to Implant Contact (BIC)

Like new bone formation, the BIC grew significantly over time and asymptotically converged to a maximum value in all three experimental groups (see Table 3, “Time model”).

In comparison to the control group (acrylates), the vinylester group showed significantly less (see Table 3, negative estimates of −0.71 and −0.86) bone to implant contact in univariate and multivariate analysis. This result corresponds with histology but is worse than new bone formation measured in this group. The obvious lack of sufficient bone to implant contact accompanied by average to good new bone formation may underline the inhibiting effect of the high amount of hydrophobic 4VE within the vinylester containing resin [16].

The results from the vinylcarbonates differ distinctly as they show significantly more BIC than the control group in univariate and multivariate analysis of the regression model (see Table 3, “Photopolymer model”). This statistical observation reflects the histologic growth pattern of newly formed bone in the vinylcarbonate group, showing long distance areas of tight bone to implant contact (see Figure 5g–i).

**Table 3:** Poisson regression models of bone to implant contact (BIC) estimated by photopolymer, time, and both. The first column shows the regressors, the second the estimated coefficients of the univariate models, the third the *p*-values of the univariate Wald’s tests, the fourth the estimates of the multivariate model (BIC = e^intercept + Vinylesters + Vinylcarbonates + 1/time^), and the last column the *p*-values of the Wald’s test of the multivariate model.

Factor	Univariate Estimate [95% CI]	*p*-value of Univariate Model	Multivariate Estimate [95% CI]	*p*-value of Multivariate Model
*“Photopolymer model” of bone to implant contact*				
Intercept (control) Acrylates	2.35 [2.03; 2.64]	<0.001	3.00 [2.61; 3.38]	<0.001
Vinylesters	−0.71 [−1.26; −0.20]	0.008	−0.86 [−1.41; −0.34]	0.002
Vinylcarbonates	1.20 [0.88; 1.54]	<0.001	0.95 [0.63; 1.29]	<0.001
*“Time model” of bone to implant contact*				
Intercept	3.68 [3.40; 3.98]	<0.001		
1/time	−37.77 [−55.38; −22.41]	<0.001	−24.51 [−41.60; −12.21]	0.001

## 4. Conclusions

To circumvent shortcomings of widely used polymers based whether on poly(lactic acid) or (meth)acrylates, we investigated two previously developed biophotopolymer classes, that are chemically based on non-toxic poly(vinyl alcohol). We successfully compared these vinylesters and vinylcarbonates *in vitro* and *in vivo* to standard acrylates.

*In vitro*, both vinylester and vinylcarbonates showed superior results to acrylates by testing monomer toxicity and alkaline activity on MC3T3-E1 cells and therefore proved the suitability of the new monomers for biomedical application.

*In vivo*, polymerized 3D cellular structures containing vinylesters showed similar rates of new bone formation but significantly less bone to implant contact, when compared to acrylates. This lack of bone to implant contact may be associated with a high amount of hydrophobic monomers within the resin. As a consequence, the monomer formulation of these vinylesters should be adapted and improved [22].

The investigated vinylcarbonates showed the best overall results in this trial setting. Besides excellent *in vitro* behavior of vinylcarbonate monomers, *in vivo* application revealed superior rates of new bone formation, bone to implant contact, and signs of polymer degradation. These findings underline the promising role of vinylcarbonate based biophotopolymers for future biomedical applications [8,17].
